# Extrapyramidal plasticity predicts recovery after spinal cord injury

**DOI:** 10.1038/s41598-020-70805-5

**Published:** 2020-08-24

**Authors:** E. Huber, R. Patel, M. Hupp, N. Weiskopf, M. M. Chakravarty, P. Freund

**Affiliations:** 1 Spinal Cord Injury Center Balgrist, University Hospital Zurich, Zurich, Switzerland; 2 Computational Brain Anatomy Laboratory (CoBrA Lab), Douglas Research Centre, Montreal, QC Canada; 3 Department of Biological and Biomedical Engineering, McGill University, Montreal, QC Canada; 4 Department of Psychiatry, McGill University, Montreal, QC Canada; 5 Wellcome Trust Centre for Neuroimaging, UCL Institute of Neurology, University College London, London, UK; 6 Department of Brain Repair and Rehabilitation, UCL Institute of Neurology, University College London, London, UK; 7 Department of Neurophysics, Max Planck Institute for Human Cognitive and Brain Sciences, Leipzig, Germany; 8 Felix Bloch Institute for Solid State Physics, Faculty of Physics and Earth Sciences, Leipzig University, Linnéstraße 5, 04103 Leipzig, Germany

**Keywords:** Biophysical models, Spinal cord diseases

## Abstract

Spinal cord injury (SCI) leads to wide-spread neurodegeneration across the neuroaxis. We explored trajectories of surface morphology, demyelination and iron concentration within the basal ganglia-thalamic circuit over 2 years post-SCI. This allowed us to explore the predictive value of neuroimaging biomarkers and determine their suitability as surrogate markers for interventional trials. Changes in markers of surface morphology, myelin and iron concentration of the basal ganglia and thalamus were estimated from 182 MRI datasets acquired in 17 SCI patients and 21 healthy controls at baseline (1-month post injury for patients), after 3, 6, 12, and 24 months. Using regression models, we investigated group difference in linear and non-linear trajectories of these markers. Baseline quantitative MRI parameters were used to predict 24-month clinical outcome. Surface area contracted in the motor (i.e. lower extremity) and pulvinar thalamus, and striatum; and expanded in the motor thalamus and striatum in patients compared to controls over 2-years. In parallel, myelin-sensitive markers decreased in the thalamus, striatum, and globus pallidus, while iron-sensitive markers decreased within the left caudate. Baseline surface area expansions within the striatum (i.e. motor caudate) predicted better lower extremity motor score at 2-years. Extensive extrapyramidal neurodegenerative and reorganizational changes across the basal ganglia-thalamic circuitry occur early after SCI and progress over time; their magnitude being predictive of functional recovery. These results demonstrate a potential role of extrapyramidal plasticity during functional recovery after SCI.

## Introduction

Spinal cord injury (SCI) leads to permanent functional deficits below the level of injury. Neurorehabilitation can foster sensorimotor recovery, but often only partial improvements can be achieved. Recovery is paralleled by a cascade of progressive neurodegenerative changes affecting the pyramidal, sensory and limbic system^1,2^; its magnitude predicting functional recovery^1,2^. In addition, compensatory changes within the extrapyramidal system might contribute to recovery as shown in the non-human primate model of SCI^3,4^. For example, impaired information flow within the basal ganglia-thalamic circuit^5^ and the motor cortex^6^ was associated with abnormal activation patterns and increased functional connectivity in pallido-thalamocortical loops^6^. However, in SCI patients, the role of the extrapyramidal system in sensorimotor recovery is understudied^7^.

To characterize structural trajectories of surface area and microstructural parameters, we modelled the MRI measures in terms of linear rate of change (reflecting degeneration/plasticity) and non-linear rate of change (reflecting acceleration/deceleration). In particularly, T1-weighted volumes were used to track trajectories of vertex-wise surface area contractions and expansions of the basal ganglia and thalamic subnuclei over 2-years. Myelin-sensitive magnetization transfer saturation (MT) and longitudinal relaxation rate (R1), a well as iron-sensitive effective transverse relaxation rate (R2*)^8,9^ were used to characterize associated microstructural changes within the same regions. Finally, early MRI markers of basal ganglia and thalamic subnuclei changes at 1-month post-SCI were used to predict recovery at 2-years.

## Methods

### Subjects

17 SCI patients were recruited at the Balgrist University Hospital and 21 healthy controls from the local neighborhood between July 2010 and July 2016. All patients had a subacute SCI (< 3 months) without brain injury, and all participants had no pre-existing neurological, mental or medical disorders affecting functional outcome. A subset of this cohort was reported previously^10–13^.

All participants underwent a comprehensive MRI protocol. In total, 182 appointments were attended by 38 participants (17 SCI patients, 21 healthy controls), at baseline (1-month post-SCI) and at 3, 6, 12, and 24 months post-SCI. Follow-ups were performed successfully in 82.4%, 100%, 100% and 76.5% of the patients, respectively in 100%, 100%, 95.2% and 100% of the controls. At the same time points, patients were also clinically assessed including 1) the International Standards for Neurological Classification of Spinal Cord Injury (ISNCSCI) protocol for motor, light-touch and pin-prick score, and 2) the Spinal Cord Independence Measure (SCIM) to measure daily life independence. The mean age between patients (mean age 43.8 years) and controls (mean age 35.7) did not differ (Mann–Whitney test, p = 0.12).

The study protocol was in accordance with the Declaration of Helsinki and was approved by the local ethics committee of Zurich “Kantonale Ethikkommission Zurich, KEK” (EK-2010–0,271). All participants provided written informed consent before participation.

### Image acquisition

Participants were scanned with a 3 T Magnetom Verio or the upgraded Skyra Fit MRI scanner (Siemens Healthcare, Erlangen, Germany) operated with a 16-channel radiofrequency receive head and neck coil and radiofrequency body transmit coil.

At each time point, the MRI measurement consisted of two protocols: (1) an optimized 3 dimensional high-resolution T1-weighted Magnetization Prepared Rapid Acquisition Gradient-Echo (MPRAGE) sequence^14^ was used to segment the basal ganglia structures, and (2) a multi-parametric mapping protocol based on multi-echo 3D FLASH sequences^15^ was used to extract underlying microstructural parameters.

For the T1-weighted MPRAGE sequence, 176 sagittal partitions were acquired with a resolution of 1 mm isotropic within a nominal scan time of 9.04 min (field of view (FOV) = 224 × 256mm^2^, repetition time (TR) = 2420 ms, echo time (TE) = 4.18 ms, inversion time = 960 ms, flip angle (FA) = 9°, readout bandwidth = 150 Hz/pixel).

The MPM protocol consisted of 3 volumes including a T1-weighted image (TR = 25 ms, FA = 23°), a proton density (PD)-weighted image (TR = 25 ms, FA = 4°), and a magnetization transfer (MT)-weighted image (TR = 37 ms, FA = 9°) with 1 mm isotropic resolution. Total scan time was 23 min (FOV = 240 × 256mm^2^, readout bandwidth = 480 Hz/pixel) (for further details see^10–13^).

### Image analysis

#### Segmentation of basal ganglia based on T1-weighted MPRAGE images

Thalamus, striatum, and globus pallidus were automatically identified using a the MAGeT Brain algorithm^16,17^. In this approach, an a priori defined atlas is first registered to a representative subset of the dataset (21 subjects) using nonlinear transformations^18^. This set of subjects then serves as a set of templates and all other subjects are warped to these templates using non-linear registration, resulting in 21 candidate segmentations for each subject. Nonlinear registration was performed using a version of Automatic Normalization Tools (ANTs) registration that is compatible with the minc file format (https://github.com/vfonov/mincANTS). After data quality assessment of all attended scans, this resulted in a total number of MPRAGE scans analyzed of 20/17 (controls/patients) at baseline, 21/14 at 3-month, 21/17 at 6-month, 20/17 at 12-month and 21/13 at 24-month.

#### Vertex-wise surface area estimation

A quantitative surface-based methodology^16^ was used to estimate the shape of each structure at study. Atlas to subject transformations were applied to 3D surface models of each labelled atlas structure, resulting in 21 candidate surfaces for each subject. The final surface was developed by computing the median location of each vertex across the 21 candidate surfaces. Second, transformations from the 21 candidate segmentations were averaged within each subject and then across the template library to reduce noise^16^. Then, for each vertex, a third of the surface of each connecting triangle is assigned to the vertex surface area. A surface-base diffusion-smoothing kernel was then applied (5 mm and 3 mm for the thalamus/striatum and globus pallidus, respectively).

#### Microstructural analysis

MT-weighted, PD-weighted, and T1-weighted volumes were used to estimate quantitative MT, R1, and R2* maps as described previously^15^. R1 maps were corrected for radiofrequency transmit field inhomogeneities with UNICORT^15^. For each subject and time point, MT, R1, and R2* maps were coregistered to their respective T1-weighted image using a 6 parameter registration with a normalized mutual information cost function (https://github.com/CobraLab/minc-toolkit-extras/blob/master/bestlinreg_g). After coregistration, segmentations of the thalamus, striatum, and globus pallidus (including the subnuclei) obtained from MAGeT Brain were used as a binary mask to extract mean values of MT, R1, and R2* for each subject and time point.

The MPM protocol was not acquired in 3 controls at any time point and in 3 patients/1 healthy control at baseline, 6 patients at 3-month, 1 patient at 6-month, and in 4 patients at 24-month. After data quality assessment, this results in a total number of scans analyzed of 17/12 (patients/controls) at baseline, 11/17 at 3-month, 16/18 at 6-month, 17/17 at 12-month, and 13/18 at 24-month.

### Statistics

R (R core team, 2015) was used for all statistical analysis. Clinical measures and vertex-wise shape measures were analyzed using mixed-model regression as it permits the inclusion of multiple measurements per person at different time points and at irregular intervals between measurements. We first compared rates of change of these measures between patients and healthy controls by including a group × time (reflecting degeneration/plasticity) and a group x squared time (reflecting acceleration/deceleration) interaction. For clinical measures, time was modelled on a log scale to account for non-linear recovery patterns. Age and gender were included to accommodate potential confounding effects of no interest. We also performed a within patient analysis to look at rates of change of vertex wise measurements between cervical and thoracic injuries. To do this, we performed a similar analysis to that described above, but with grouping being either cervical or thoracic as opposed to patient vs. control. To identify in which specific nuclei group differences were observed, we superimposed surface-labels of the cytoarchitectonic atlas for the thalamus^19^ on our thalamic template. Second, in those structures that showed group differences in surface area over time, we explored underlying differences in microstructure (R1, MT, R2*) using the same models. Associations between quantitative MRI parameters at 1-month (baseline) and clinical recovery at 2-years were investigated using linear regression models adjusted for age, gender and clinical baseline scores. All vertex-wise analyses were corrected for multiple comparisons using the false discovery rate (FDR) at 10%.

## Results

### Demographics

We included a total of 17 SCI patients and 21 healthy controls. The mean age of patients did not differ from controls (p > 0.05). The baseline scan was acquired 1.4 ± 0.5 months after injury and the remaining scans at 3.5 ± 1.5, 7.1 ± 1.9, 13.3 ± 3.1 and 28.7 ± 5.1 months. Over 2-years, patients recovered by 2.76 points per log month on their lower extremity motor score [p = 0.001, 95% CI (1.07 4.45)], by 0.91 point per log month on their upper extremity motor score [p = 0.046, 95% CI (0.02 1.80)], and by 7.89 points per log month on their SCIM score (p < 0.001, 95% CI (5.03 10.75)) (Table 1). Light-touch and pin-prick score remained unchanged over time (p = 0.152, p = 0.790). For subject wise plots of trajectory of each clinical score, see Supplementary Information (Clinical Trajectories).

**Table 1:** Clinical and epidemiological data for all patients included in the study.

ID	Age at injury	Initial Site of neurological Impairment (motor/sensory)	INSCSCI upper extremity motor score (Baseline/24-months)	INSCSCI lower extremity motor score (Baseline/24-months)	INSCSCI light-touch score (Baseline/24-months)	INSCSCI pin-prick score (Baseline/24-months)	SCIM score (Baseline/24-months)
p1	18	C5/C4	19/22	0/0	24/30	27/32	4/22
p2	23	C7/C6	42/48	0/23	69/70	38/33	23/70
p3	68	T10/T10	50/50	2/31	78/74	75/65	41/46
p4	44	T12/T12	50/50	39/45	107/106	109/98	84/100
p5	42	C6/C6	23/26	0/0	27/21	20/17	18/41
p6	71	C7/C8	36/48	16/42	85/66	36/25	17/43
p7	20	C5/C5	21/21	0/0	21/45	19/18	4/39
p8	30	C7/C8	47/47	0/0	64/72	35/30	38/40
p9	52	T9 /T9	50/50	48/49	95/94	89/90	84/100
p10	42	C5/C4	41/36	46/48	104/99	104/96	99/100
p11	29	T11/T11	50/50	10/12	86/76	88/69	52/68
p12	71	T1/T10	50/50	43/50	88/93	72/67	47/69
p13	71	T1/T4	50/50	43/50	83/112	81/112	56/97
p14	73	T1/T11	50/50	0/0	75/71	77/72	28/36
p15	31	C4/C5	20/26	0/0	22/31	19/20	13/25
p16	28	C4/C3	15/10	0/0	14/23	14/23	16/20
p17	32	T1/T4	50/50	9/50	79/112	58/77	40/96

*INSCSCI* International Standards for Neurological Classification of Spinal Cord Injury; *SCIM* Spinal Cord Independence Measure.

### Time course of thalamic changes

**Figure 1:**
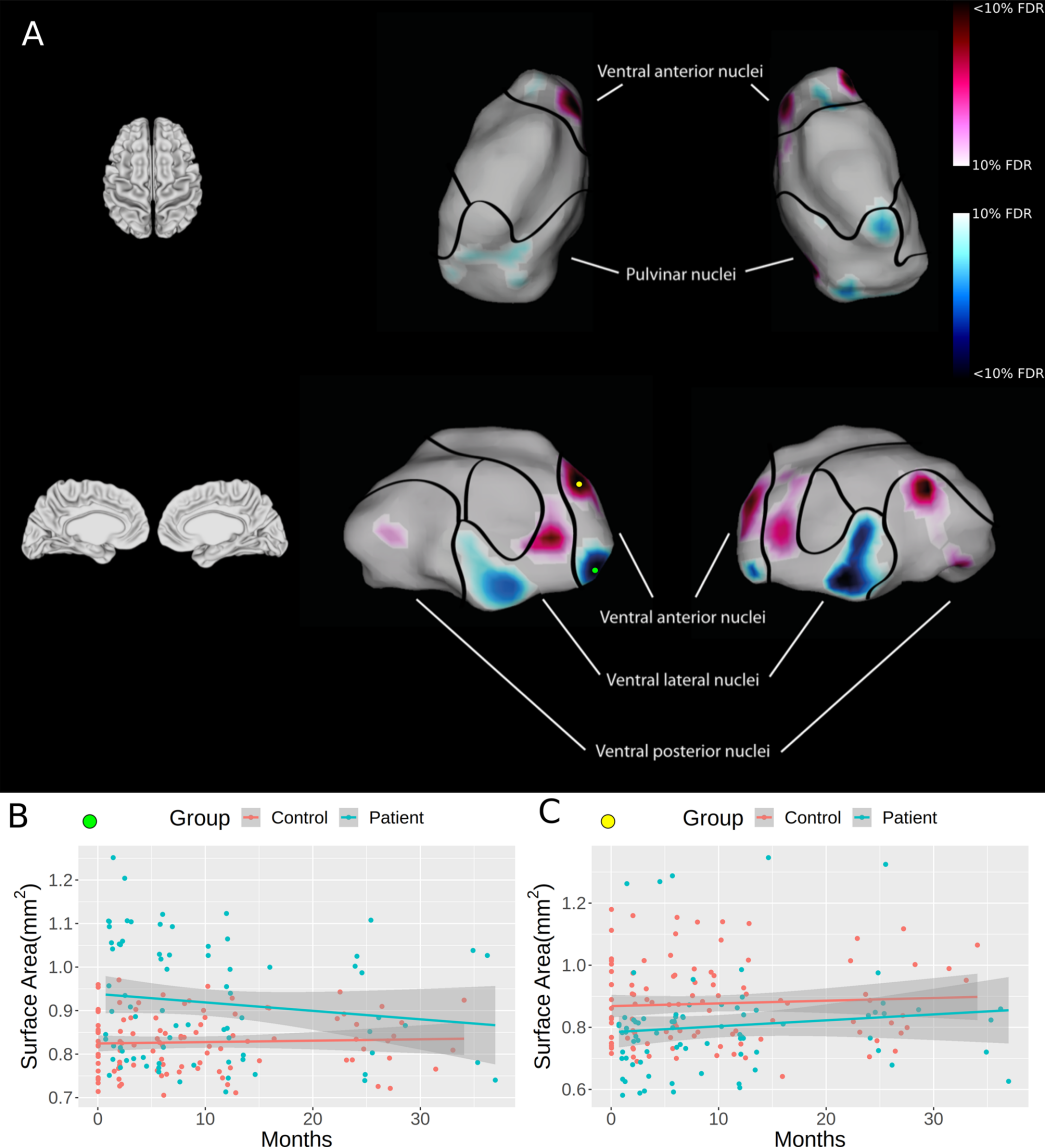
Linear shape differences within the thalamus estimated as surface area contractions and expansions. All surface area contractions (representing degeneration) are represented in blue, and all surface area expansions (reflecting compensatory plasticity) are shown in red. Compared to controls, patients showed contractions of surface area within the inferior part of the ventral anterior and ventral lateral nuclei (motor nuclei) and within the pulvinar nuclei over the first 2-years after spinal cord injury. Surface area expansions were evident in the superior parts of the ventral anterior and ventral lateral nuclei (motor nuclei), as well as within the ventral posterior nuclei (sensory nuclei). Note that the borders of the thalamic subnuclei were identified using the atlas of Chakravarty et al. (2006). Colour bars denote the FDR threshold applied in both the positive (red, expansion) and negative (blue, contraction) directions. Plots B and C denote the surface area measurements across time at peak vertices denoted by the green (B) and yellow (C) markers, illustrating the different group trajectories.

Both thalami showed a greater linear rate in surface area contractions in patients compared to controls over time within the inferior parts of the ventral anterior nuclei (VAN), and surface expansion within its superior parts (Fig. 1, Suppl. Table 1). In the left thalamus, these changes were levelling-off over time (Suppl. Fig. 1). In the ventral lateral nuclei (VLN), linear surface contractions were observed in its inferior parts, which levelled-off over time in the right nuclei. Greater linear rate in surface area expansions were found within the superior parts of the VLN in patients compared to controls over time. Greater linear rate in surface area expansions was evident within the ventral posterior nuclei (VPN) in patients compared to controls over time, which rate of change decelerated within the right VPN. Both pulvinar nuclei showed greater rate in linear surface contractions in patients compared to controls, in particular within the lateral and the medial pulvinar. Microstructural changes are summarized in Table 2. No significant difference in the rate of change were observed between patients with cervical or thoracic injuries.

**Table 2:** Microstructural changes.

	R1 (s^−1^)		MT (p.u.)		R2* (s^−1^)	
	Mean/(95% CI)	p =	(mean/95% CI)	p = p =	(mean/95% CI)	p =
**Linear changes over time per month**						
Thalamus						
VAN						
Left	− 6.527 (− 11.526 to 1.527)	0.011				
VLN						
Left	− 6.054 (− 11.018 to 1.089)	0.017				
Right	− 5.156 (− 9.760 to 0.552)	0.028				
VPN						
Left	− 5.185 (− 10.214 to 0.157)	0.043				
Striatum						
Ventral striatuml						
Left			− 0.020 (− 0.039 to 0.001)	0.047		
Right			− 0.017 (− 0.033 to 0.001)	0.037		
Pre-commissural caudate						
Left	− 4.598 (− 8.887 to 0.308)	0.036	− 0.020 (− 0.038 to 0.003)	0.021		
Post-commissural caudate						
Left					− 0.001 (− 0.001 to 0.001)	0.034
Globus pallidus						
Left	− 5.570 (− 10.728 to 0.411)	0.034				
Right	− 5.674 (− 10.763 to 0.585)	0.029				
**Acceleration and Deceleration per month**^**2**^						
Thalamus						
VAN						
Left	0.193 (0.032 0.354)	0.019				
VLN						
Left	0.173 (0.0131 0.333)	0.034				
Right	0.159 (0.011 0.307)	0.036				
Striatum						
Pre-commissural caudate						
Left	0.164 (0.025 0.303)	0.021				
Globus pallidus						
Left	0.178 (0.013 0.343)	0.034				
Right	0.187 (0.024 0.350)	0.024				

Rates of changes of myelin-sensitive R1, MT and R2* in patients compared to controls over time. Negative numbers indicate linear decreases, respectively acceleration, in patients compared to controls, whereas positive values indicate linear increases, respectively deceleration.

### Time course of striatal changes

**Figure 2:**
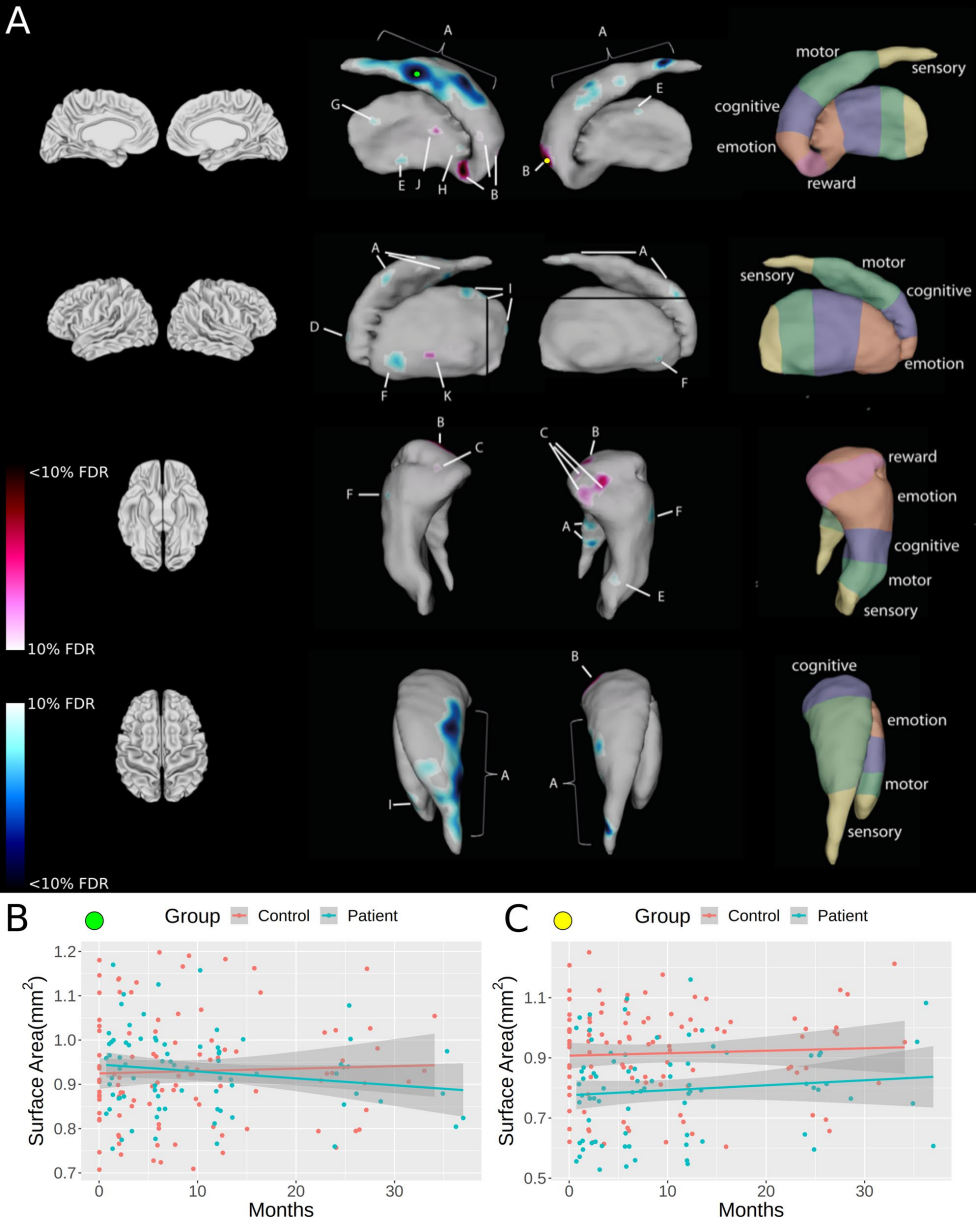
Linear shape differences within the striatum estimated as surface area contractions and expansions. All surface area contractions (representing degeneration) are represented in blue, and all surface area expansions (reflecting compensatory plasticity) are shown in red. Compared to controls, patients showed contractions of surface area within the putamen, and the caudate, in particular in regions involved in sensorimotor and emotion processing, and in cognitive function over the first 2-years after spinal cord injury. Surface area expansions were evident in regions involved in cognition, emotion and reward processing. Note that the atlas on the right shows the somatotopy of the striatum, based on a review of functional MRI studies (Arsalidou et al. 2013). Labels therefore represent approximate regions of corresponding functions. Colour bars denote the FDR threshold applied in both the positive (red, expansion) and negative (blue, contraction) directions. Plots B and C denote the surface area measurements across time at peak vertices denoted by the green (B) and yellow (C) markers, illustrating the different group trajectories.

In the striatum, patients showed bilateral surface area contractions and expansions over time in the caudate and the putamen compared to controls. Specifically, greater linear rate in surface area contractions were evident medially within the central to posterior part of the bilateral caudate (Fig. 2A), which levelled-off over time in patients compared to controls (Suppl. Fig. 2). Greater linear rate in expansions of surface area were observed in the bilateral inferior part of the anterior caudate (Fig. 2B), bilaterally levelling-off over time. Greater linear rate in expansions of surface area was observed in the inferior parts of the ventral caudate (i.e. nucleus accumbens; Fig. 2C), which showed a levelling-off over time at the left side only. Greater linear rate of surface area contractions was evident in the left lateral part of the anterior caudate (Fig. 2D). Within the putamen, greater rate of liner contractions of surface area were observed in the medial central (Fig. 2E) as well as the lateral anterior putamen, bilaterally (Fig. 2F). The surface area of the right lateral anterior putamen showed a levelling-off over time in patients compared to controls. In addition, surface area contractions were observed in the left medial posterior (Fig. 2G), left medial anterior (Fig. 2H) and left lateral superior posterior putamen (Fig. 2I). Expansions in surface area were detected within the left medial anterior (Fig. 2J) and lateral anterior putamen (Fig. 2K). Expansions within the left anterior putamen levelled-off over time in patients compared to controls. Microstructural changes are summarized in Table 2. No significant difference in the rate of change were observed between patients with cervical or thoracic injuries.

### Time course of pallidal changes

The surface area analysis demonstrated no changes over time in patients compared to controls in the globus pallidus. Microstructural changes are summarized in Table 2. No significant difference in the rate of change were observed between patients with cervical or thoracic injuries.

### Relationship of MRI parameters at 1-month post-SCI and clinical recovery at 2-years

**Figure 3:**
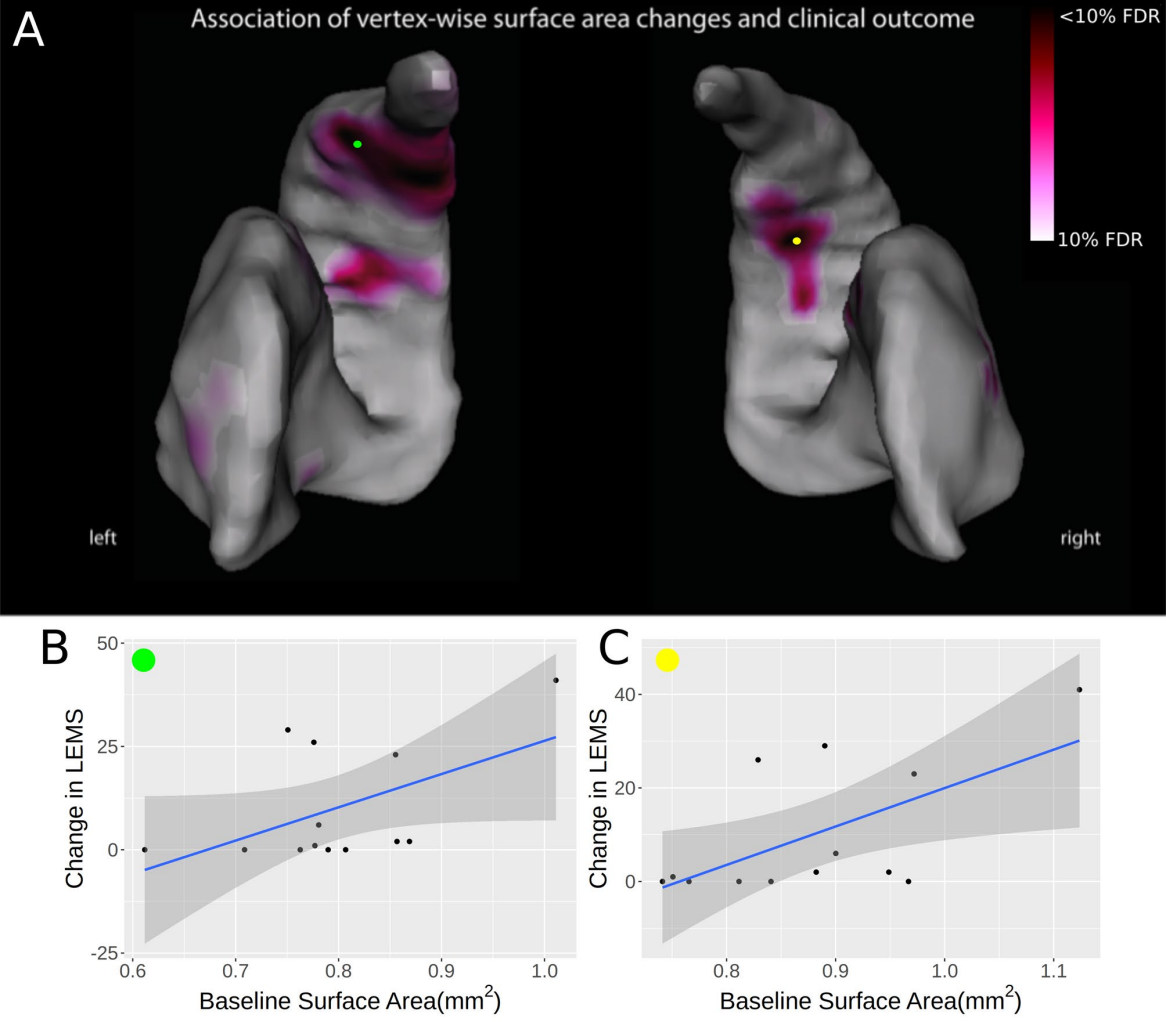
Relationship between 1-month structural and microstructural parameters and recovery at 2-years. Local morphological features of the striatum (motor region) at 1-month after spinal cord injury were associated with better lower extremity motor score at 2-years, adjusted for age, gender and baseline clinical score. (**A**) Vertices in red show a relationship of surface area expansions (reflecting compensatory plasticity) with increased lower extremity motor score at 2-years, corrected at 10% FDR. For each of left (**B**) and right (**C**) striatum we plot surface area versus change in lower extremity motor score for a given vertex, denoted by green and yellow markers respectively. For the left striatum vertex, correlation was 0.52 (95% CI [− 0.03, 0.83]). For the right striatum, correlation was 0.61 (95% CI [0.096, 0.87]).

Through running linear models at each vertex, we found that surface area measurements at baseline were significantly related to lower extremity motor score at the 2-year timepoint. Specifically, a cluster of voxels showing increased surface area expansions within the central caudate and the lateral central-posterior putamen at 1-month were associated with better lower extremity motor score at 2-years, adjusted for baseline score (Fig. 3).

## Discussion

This study characterizes neurodegenerative and reorganizational changes across the basal ganglia-thalamic circuit after SCI using advanced neuroimaging measures, in vivo. We demonstrate a dynamic pattern of neurodegenerative and reorganizational changes in the basal ganglia-thalamus circuitry within the first 2-years post-SCI, its magnitude predicting functional recovery. These observations illustrate the enduring neurodegenerative and neuroplastic processes induced by a SCI and highlight a progressive diaschisis across the neuroaxis.

### Time course of thalamic changes

We have previously shown in the same patient cohort that a focal injury to the spinal cord produces neurodegenerative changes to the sensorimotor and limbic system^10,11,13^. Moreover, the atrophic thalamus showed also an accumulation of iron content, however the exact location of these changes was not identified^13,20^. This study shows that SCI leads to bilateral surface area contractions in the inferior parts of the VAN and VLN, while surface area expansions were evident in the superior parts of the VAN, VLN and VPN. The changes in all of these nuclei were paralleled by decreases in myelin-sensitive R1 over time.

Both, the VAN and VLN receive input from the globus pallidus and project to the premotor and motor cortex^21^. In contrast, the VPN receives input from the lemniscus medialis (transmitting light touch, vibration, and proprioception) and from the spinothalamic tract (transmitting pain and heat sensation)^21^. All of these nuclei are somatotopically organized^21^ with distal muscles represented dorsal to those for proximal limb portions; and more cranial representations were found more anterior^22^. This suggests that the contracting surface area (i.e. local volume decreases) within the lower limb representing areas in motor thalamic nuclei are likely neurodegenerative changes while surface area expansions (i.e. local volume increases) in upper limb representing areas of sensorimotor thalamic nuclei could be interpreted as compensatory changes on the assumption that performance improvements during training^23,24^ or neurorehabilitation^24,25^ induces grey matter volume increase.

Possible biological substrates for local volume decreases (i.e. surface area contractions) might include changes in the number of neurons induced by trans-synaptic degeneration, neuronal death, demyelination, synaptic pruning, or changes in the number of glial cells^26^. Axonal sprouting and activity-dependent plasticity^27^ might account for volume increases (i.e. surface area expansions). In particular, compensatory use of the upper limbs might drive expansion of cortical M1 hand area into output-deprived leg area^28^ as a consequence of the rewired hind limb neurons onto cervical motor circuits^28^; the same mechanism might also be occurring in the basal ganglia. However, surface area changes are not specific to any single pathological process. Here we show that changes in myelin-sensitive R1^29^ values might be interpreted as ongoing demyelination in the sensorimotor nuclei of the thalamus.

The lateral and medial pulvinar nuclei also showed surface area contractions over time. These nuclei are involved in visual attention regulation, and also coordinate sensory input with its corresponding motor response^30^. As most SCI patients have motor and sensory deficits, it seems likely that the coordination between these two modalities is also altered^31^. In addition, SCI patients rely more on their visual input in order to coordinate limb movements due to impaired proprioception^32^.

### Time course of striatal changes

Surface area contractions were also observed in striatal regions involved in sensorimotor and cognition processing, and surface area expansions in regions involved in cognition, emotion and reward processing^4,33^. These changes were paralleled by decreases in myelin-sensitive R1 and MT. Interestingly, we observed bilateral surface area contraction within the medial and lateral dorsal part of the motor putamen, and as the putamen consists of two sets of somatotopic representation^34^. This indicates loss of afferents from both the primary motor cortex and the supplementary motor area. This is in line with previous studies, showing that both of these structures show altered function and structure after SCI^20,35^. Remarkably, we also observed surface area expansions in the ventral striatum (i.e. nucleus accumbens), a region known to be involved in recovery of the dexterous finger movements after experimental SCI^4,36^.

### Time course of pallidal changes

We observed decreases in myelin-sensitive R1 changes in the globus pallidus. Since the globus pallidus is the relay station between the striatum and the thalamus, trans-synaptic degeneration of afferents and efferents of the primary motor cortex and the premotor cortex may primarily lead to changes in the striatum and the thalamus.

### Effect of rostro caudal level of spinal injury on neurodegeneration

Analyzing thoracic and cervical SCI patients conjointly allows to address (i) to what extent the level of lesion and consequently the extent and kind of neurological-functional impairment are related to secondary structural changes within the extrapyramidal system, and (ii) whether some secondary effects are independent of the amount and level of spinal cord damage. As in other neurological disorders, even a very local and focal lesion can have remote effects, i.e. diaschisis)^37,38^. The fact that we did not detect a relationship between lesion level and extrapyramidal changes indicates that these remote structural changes are likely trans-synaptic and might be a general effect due to the loss of voluntary control over the limbs. This is also reflected by the association between early surface area expansion and lower limb recovery.

### Relationship of MRI parameters at 1-month post-SCI and clinical recovery at 2-years

Clinical recovery occurs most rapidly within the first 6 months and is fostered through neurorehabilitation by promoting cortical, subcortical and spinal cord neural circuit reorganisation^39,40^. Interestingly, early surface area expansions within the motor striatum were associated with better 2-year lower extremity motor score. Although all patients of the present study were admitted for neurorehabilitation within one months after the injury, there is an inherent variability (depending on lesion level and extent of impairment) of how much therapy each individual patient received already at this early timepoint. Thus it remains speculative if the expansion of surface area is indicative of compensatory plasticity—a phenomena also observed in Multiple sclerosis^41^ and Parkinson’s disease^42^ or whether it is a co-occurrence. However, our study shows the clinical viability of MRI based structural shape measures for monitoring and predicting recovery after traumatic SCI. For example these neuroimaging biomarkers could be used to assess the influence of therapeutic interventions (e.g. levodopa (L-Dopa)) on training effects druing rehabilaition not only after SCI^43^ but also stroke^44^ or other disorders where the extrapyramidal system is affected. Its future application holds promise to provide a tool to improve our understanding of the disease mechanism after SCI and might render extrapyramidal neuroimaging biomarkes. These new insights will enable us to better predict individual recovery trajectories and identify those patients who could profit from targeted interventions, such as levodopa. However, further investigations are required to assess the interplay between function, the underlying neuropathology and treatment response.

### Limitations

First, although we identified the approximate location of changes within the striatum and the globus pallidus, a histology-based atlas of the striatum and the globus pallidus is currently not available. Next, the upgrade of the MRI scanner might potentially be a confounding factor, but this was mitigated by the fact that all participants were scanned before and after the upgrade. Although the mean age of the control cohort was not significantly different from the patient cohort we included age as a (nuisance) covariate, and its inclusion made no material difference to the results (e.g. the relevant coefficient for the subject indicator terms in the repression models). Finally, the applied MPM protocol, consisting of MT, R1 and R2* measures, provides only indirect measures of myelin^45^ and iron^46^ but not specific to processes such as demyelination, degeneration and iron content.

## Conclusion

This study provides evidence for progressive extrapyramidal macro- and microstructural changes after SCI, indicating large scale, trans-neuronal remodeling of primarily unaffected brain regions. Extrapyramidal plasticity early after SCI are associated with long-term motor recovery. This study therefore provides unbiased, quantitative readouts of nuclei-specific extrapyramidal changes which are clinically eloquent.

## Supplementary information

Supplementary file1
