# Supplementary material for: Extrapyramidal plasticity predicts recovery after spinal cord injury
Source: Sci Rep. 2020 Aug 24;10:14102. doi: 10.1038/s41598-020-70805-5 (PMC7445170; doi:10.1038/s41598-020-70805-5)
Supplement: Supplementary file 1 — Supplementary file1 [file 41598_2020_70805_MOESM1_ESM.pdf]

Extrapyramidal plasticity predicts recovery after spinal cord injury

Huber E[1], Patel R[2], Hupp M[1], Weiskopf N[5, 6], Chakravarty MM[2], Freund P[1, 3, 4, 5]

[1] Spinal Cord Injury Center Balgrist, University Hospital Zurich, Zurich, Switzerland
[2] Department of Biological and Biomedical Engineering, McGill University, Montreal, QC, Canada
[3] Wellcome Trust Centre for Neuroimaging, UCL Institute of Neurology, University College London, London, United Kingdom
[4] Department of Brain Repair and Rehabilitation, UCL Institute of Neurology, University College London, London, United Kingdom
[5] Department of Neurophysics, Max Planck Institute for Human Cognitive and Brain Sciences, Leipzig, Germany
[6.] Felix Bloch Institute for Solid State Physics, Faculty of Physics and Earth Sciences, Leipzig University, Linnéstraße 5, 04103 Leipzig, Germany

Clinical Trajectories

This section contains subject wise plots showing change since baseline for each clinical score. For each score, we plotted time in months vs (score at timepoint - baseline score) and plotted results below. We also assess general group trends using linear mixed effects modelling with time in months, age, sex as fixed effects, and allowing the slope to vary per subject (eg lme(uems_~ gender_edit + age + months, data=data_patients, random=~1+months|salms, method="ML",na.action=na.exclude, control=list(opt="optim")). t and p values (uncorrected) for the months variable is specified for each clinical variable in the table below, and trajectory plots follow.

Variable	tvalue	P value
Upper extremity score	1.61	0.118
Lower extremity score	2.85	0.0059
Light touch score	0.7	0.49
Pin prick score	-1.94	0.056
SCIMTOT score	3.8	0.0003

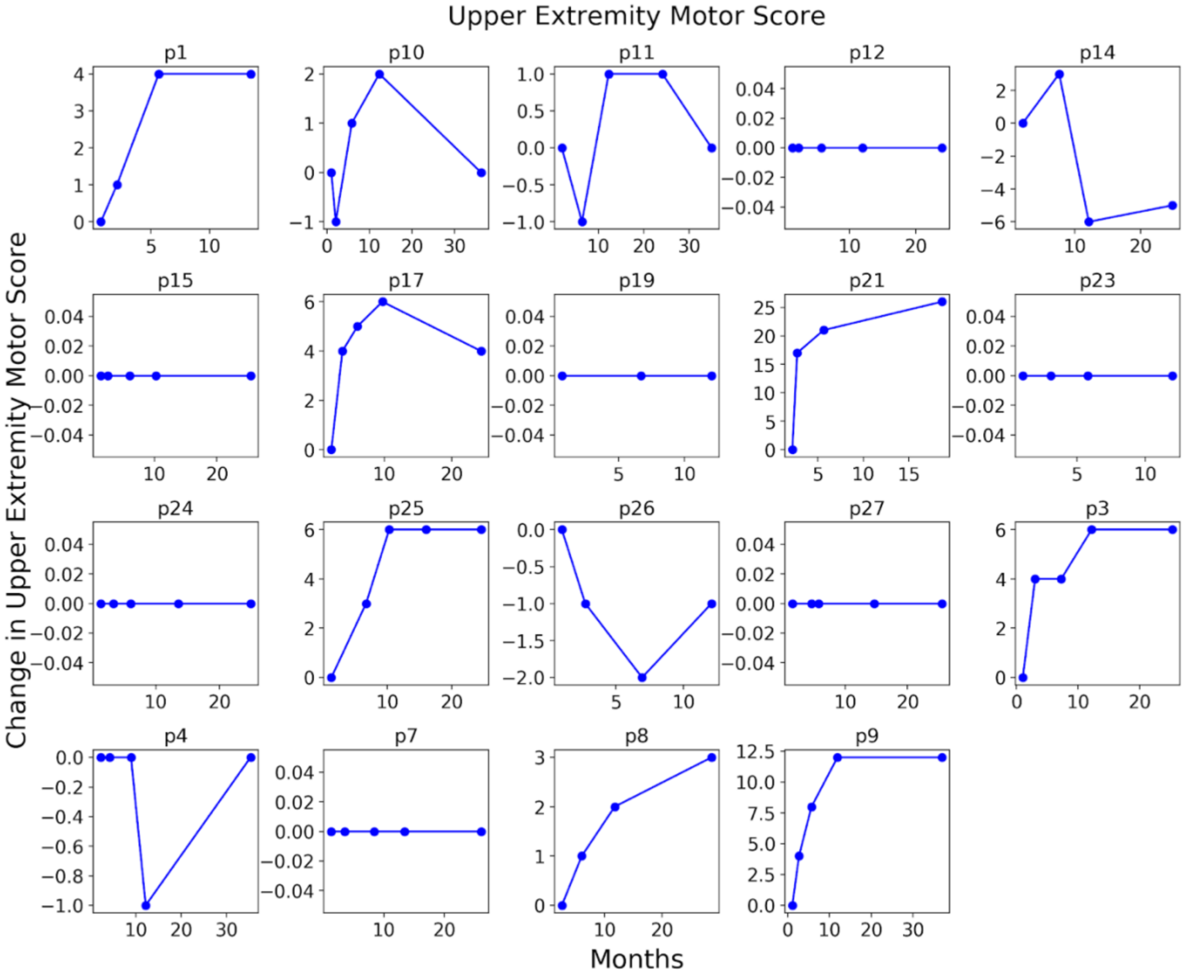
Upper Extremity Motor Score
p1
p10
p11
p12
p14
p15
p17
p19
p21
p23
p24
p25
p26
p27
p3
p4
p7
p8
p9
Change in Upper Extremity Motor Score
Months

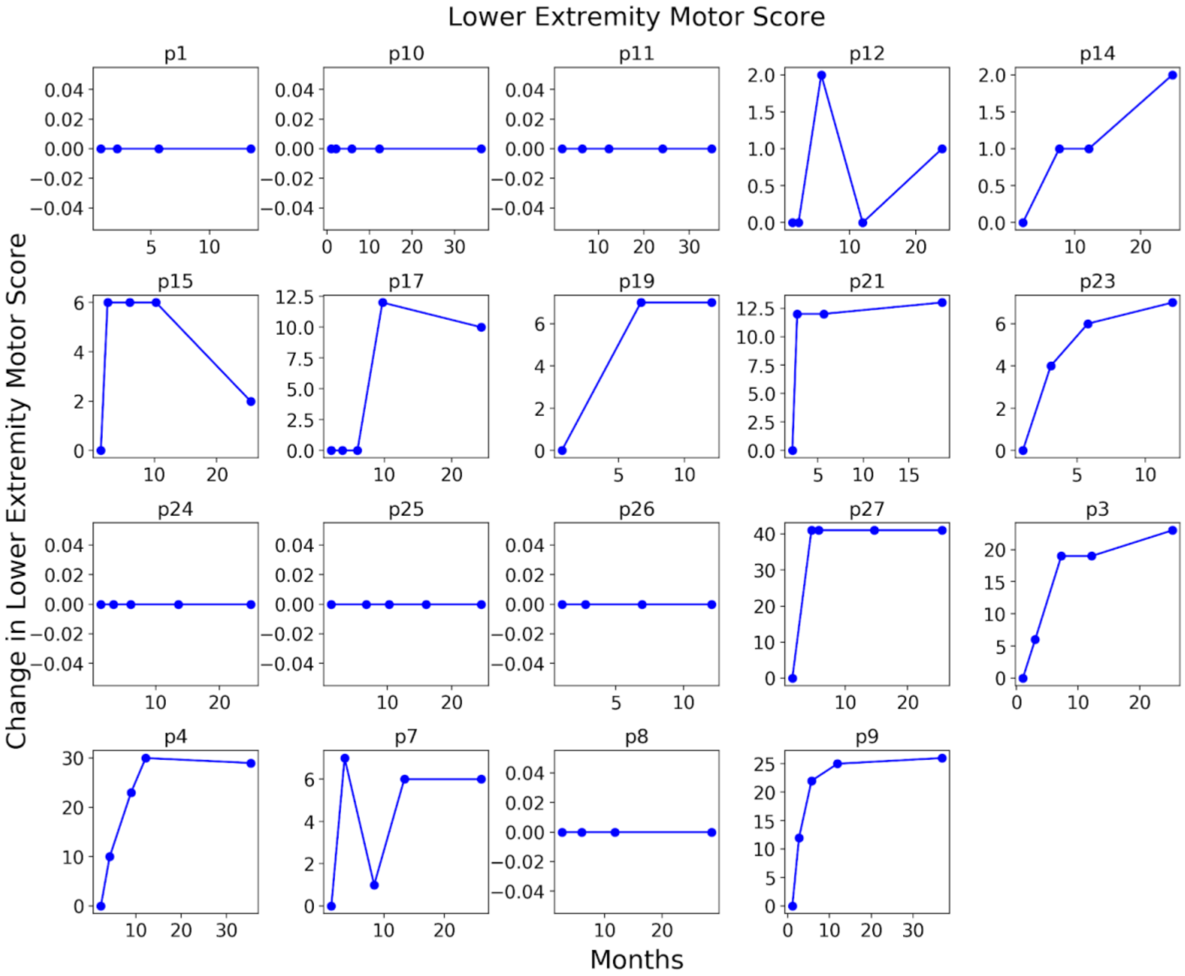

Lower Extremity Motor Score
Change in Lower Extremity Motor Score
Months
p1
p10
p11
p12
p14
p15
p17
p19
p21
p23
p24
p25
p26
p27
p3
p4
p7
p8
p9

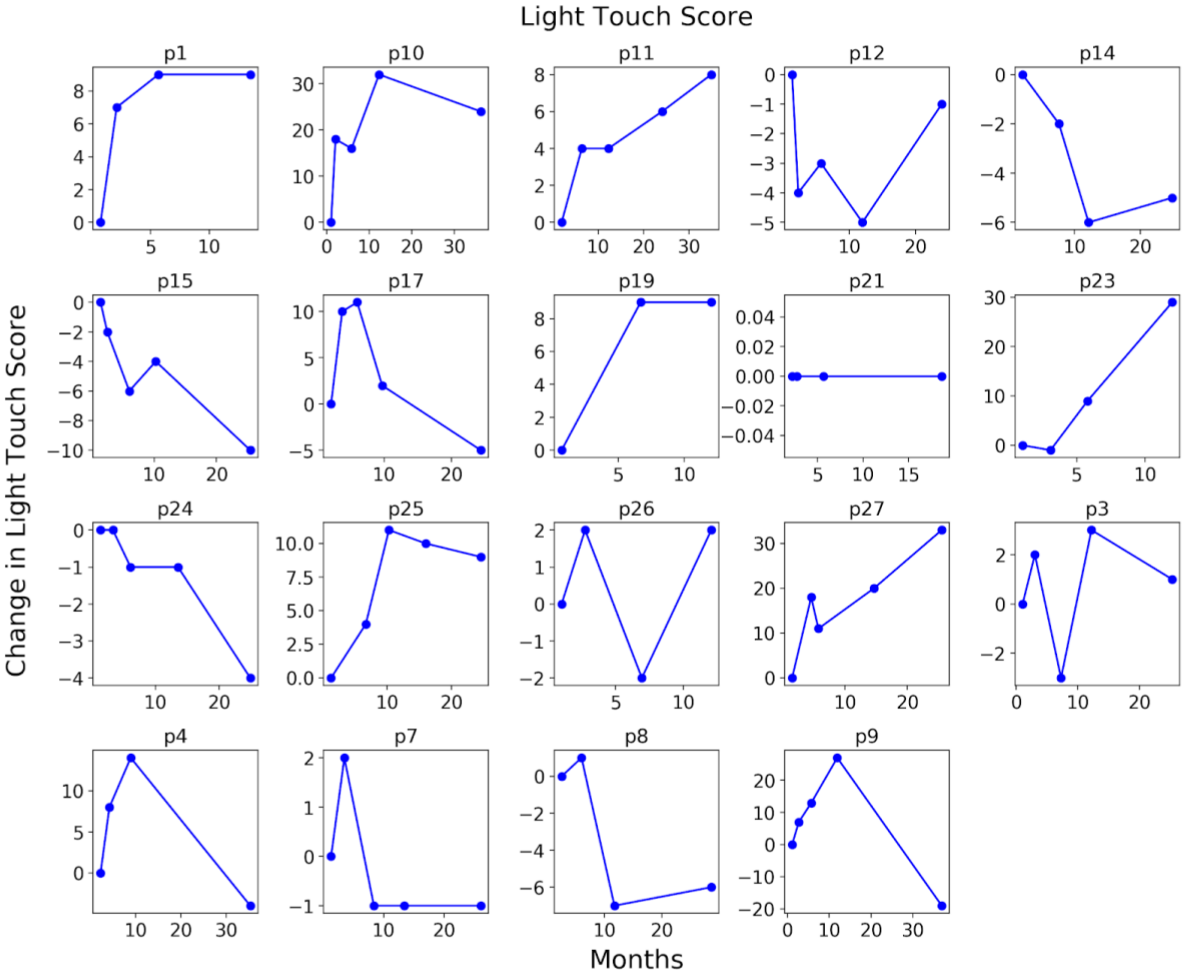

Light Touch Score
Change in Light Touch Score
Months
p1
p10
p11
p12
p14
p15
p17
p19
p21
p23
p24
p25
p26
p27
p3
p4
p7
p8
p9

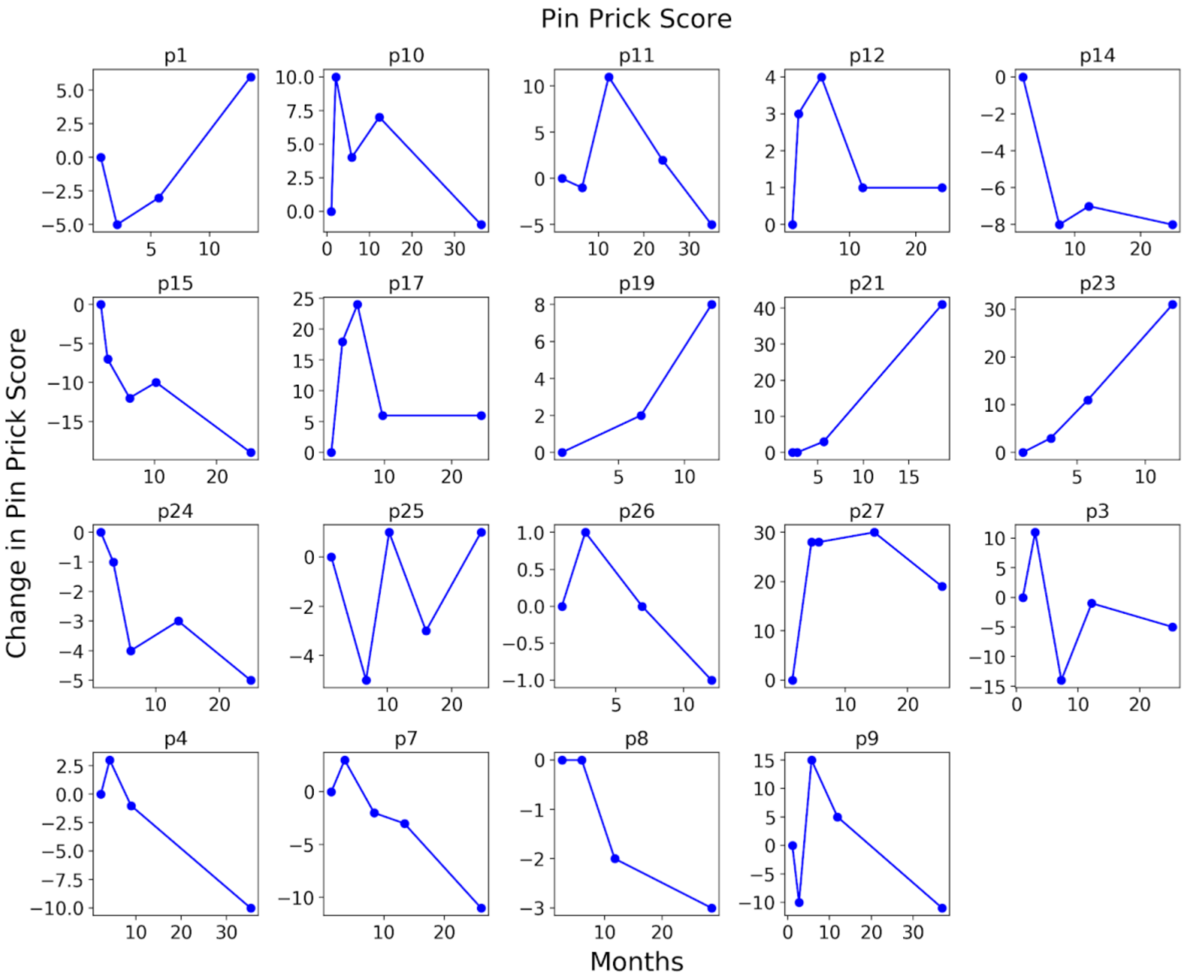
Pin Prick Score
p1
p10
p11
p12
p14
p15
p17
p19
p21
p23
p24
p25
p26
p27
p3
p4
p7
p8
p9
Change in Pin Prick Score
Months

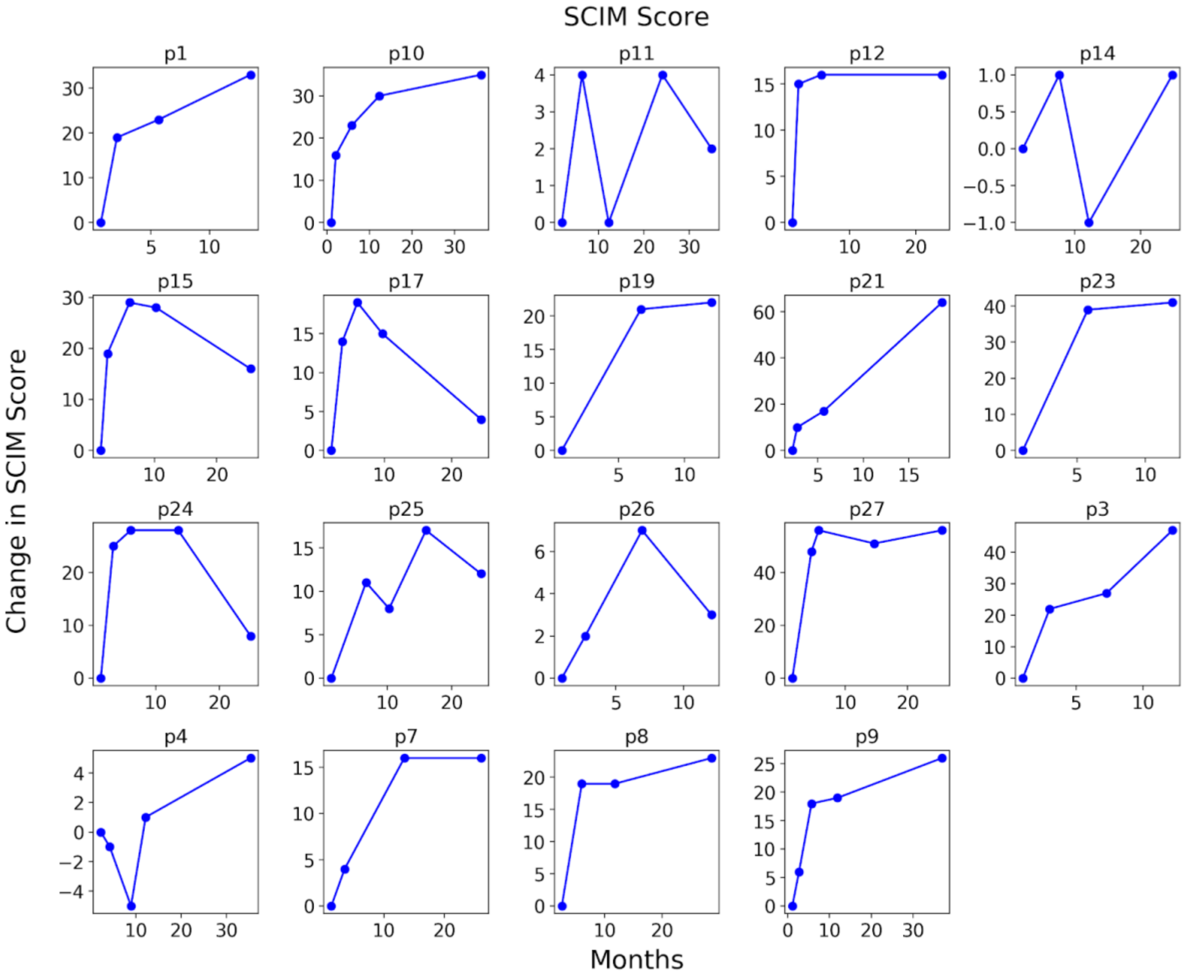
SCIM Score
p1
p10
p11
p12
p14
p15
p17
p19
p21
p23
p24
p25
p26
p27
p3
p4
p7
p8
p9
Change in SCIM Score
Months

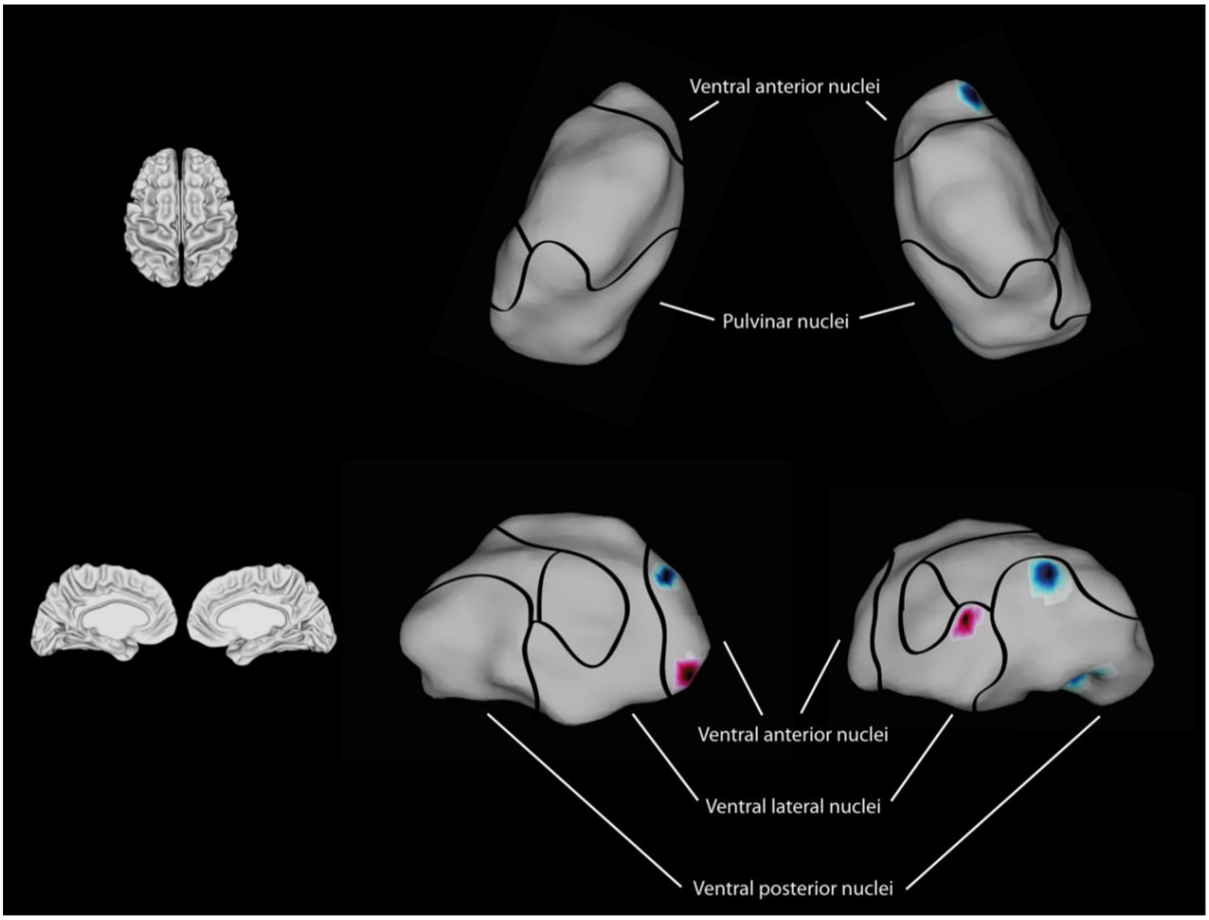

Suppl. Figure 1: Non-linear shape differences within the thalamus estimated as significant surface area contractions and expansions. All surface area contractions are represented in blue color, and all surface area expansions are shown in red color (both reflecting levelling-off of linear changes). Patients showed levelling-off of surface area contractions within the left ventral anterior nucleus and within the right ventral lateral nucleus. Surface area expansions levelled-off bilaterally within the ventral anterior nuclei and within the left ventral posterior nucleus.

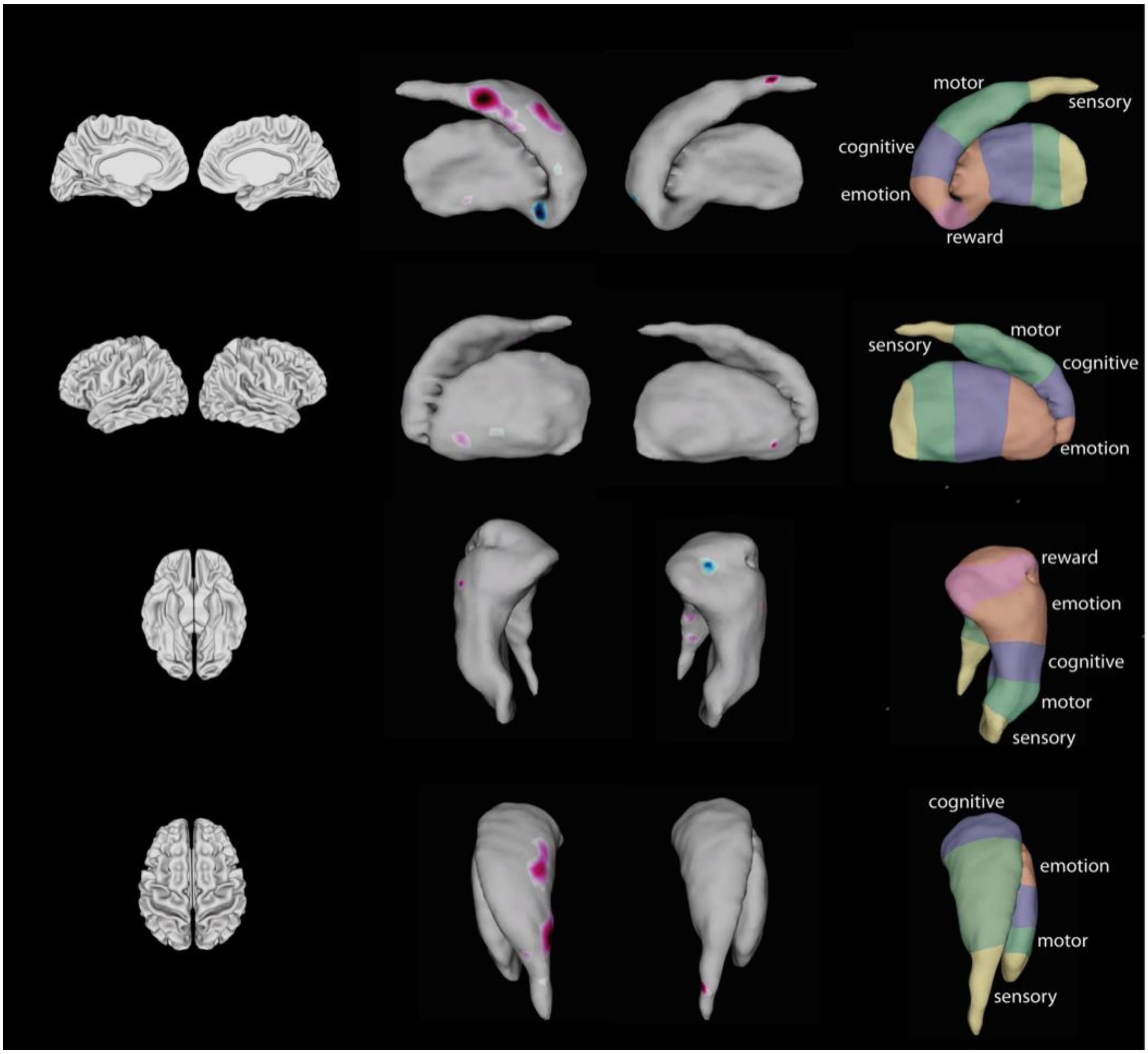

Suppl. Figure 2: Quadratic shape differences within the striatum estimated as significant surface area contractions and expansions. All surface area contractions are represented in blue color, and all surface area expansions are shown in red color (both reflecting levelling-off of linear changes). Patients showed levelling-off of surface area contractions within regions involved in sensorimotor and emotion processing over the first 24 month after SCI. Surface area expansions levelled-off in regions involved in cognition, emotion and reward. Note that the atlas on the right shows the somatotopy of the striatum, based on a review of functional MRI studies (Arsalidou *et al.*, 2013). Labels therefore represent approximate regions of corresponding functions.
